# Household food insecurity and its association with school absenteeism among primary school adolescents in Jimma zone, Ethiopia

**DOI:** 10.1186/s12889-016-3479-x

**Published:** 2016-08-17

**Authors:** Dessalegn Tamiru, Alemayehu Argaw, Mulusew Gerbaba, Girmay Ayana, Aderajew Nigussie, Tefera Belachew

**Affiliations:** 1 Department of Population and Family Health, Jimma University, P.O.Box: 378, Jimma, Ethiopia; 2 Ethiopian Public Health Institute, Addis Ababa, Ethiopia

**Keywords:** Absenteeism, Adolescents, Insecurity, Jimma

## Abstract

**Background:**

Household food insecurity and lack of education are two of the most remarkable deprivations which developing countries are currently experiencing. Evidences from different studies showed that health and nutrition problems are major barriers to educational access and achievement in low-income countries which poses a serious challenge on effort towards the achieving Sustainable Development Goals. Evidence on the link between food security and school attendance is very important to address this challenge. This study aimed to assess to what extent food insecurity affects school absenteeism among primary school adolescents.

**Methods:**

A school based cross-sectional study was conducted among primary school adolescents in Jimma zone from October-November, 2013. Structured questionnaire was used to collect data on the household food security and socio-demographic variables. Data were analyzed using SPSS for windows version 16.0 after checking for missing values and outliers. Multivariable logistic regression analyses were used to determine the association of school absenteeism and food insecurity with independent variables using odds ratio and 95 % of confidence intervals. Variables with *p* ≤ 0.25 in the bivariate analyses were entered into a multivariable regression analysis to control for associations among the independent variables.

**Results:**

The frequency of adolescent school absenteeism was significantly high (50.20 %) among food insecure households (*P* < 0.001) compared to their peers whose households were food secure (37.89 %). Findings of multivariable logistic regression analysis also showed that household food insecurity [AOR = 2.81 (1.70, 4.76)] was positively associated with poor school attendance while female-headed household [AOR = 0.23 (0.07, 0.72)], urban residence [AOR = 0.52 (0.36, 0.81)] and male-gender [AOR = 0.64 (0.54, 0.74)] were inversely associated with school absenteeism. Household food insecurity was positively associated with lack of maternal education [AOR = 2.26 (0.57, 8.93)] and poor household economic status [AOR = 1.39 (1.18, 2.83)]. However, livestock ownership [AOR = 0.17 (0.06, 0.51)] was negatively associated with household food insecurity.

**Conclusions:**

Findings of this study showed that household food insecurity has strong linkage with adolescent school absenteeism. Maternal education and household economic status were significantly associated with household food security status. Therefore, national policies and programs need to stress on how to improve family income earning capacity and socioeconomic status to handle household food insecurity which is a key contributor of adolescent school absenteeism.

## Background

Poor health and nutrition problem in schoolchildren and adolescents are major barriers to educational access and achievement in low-income countries. Studies showed that poor health and nutrition problem causes a serious challenge for the effort towards the Sustainable Development Goals (SDGs) of quality education and lifelong learning opportunities for all. Nutrition related problems are common among young adolescents in low-income countries due to their needs have been largely ignored by the nutrition policies, strategies and programs of the countries [1, 2]. Food insecurity has been shown to be especially detrimental to children’s mental and educational development. Studies showed that the problems arising from food insecure households are increased risk of academic and socio-emotional difficulties [1–3].

Household food insecurity occurs when food is not sufficiently available, cannot be accessed with certainty in socially acceptable ways, or not physiologically utilized completely [1, 3]. A wide variety of research has demonstrated the positive correlation between health and learning, and the two things are mutually reinforcing factors [3–6]. Many studies have found out that food insecurity harms children’s health in a variety of ways. Children from food-insecure households are more likely suffer from common illnesses like stomachaches, headaches, and colds when they reach preschool age [7–10].

Optimal cognitive development and physiological function in school children requires access to food in adequate quantity and quality at all points of life. Studies also showed that poor socioeconomic status and deficiency of a nutritious diet in terms of quantity and quality are mostly resulted in developmental impairments and sluggish economic developments of an individual and a country [4, 5, 8]. Food insecurity adversely affects school attendance, academic performance and social skills of young children and adolescents. Children from food insufficient households had a low grade score and were more likely to repeat a grade than their peers from food sufficient households [4, 6, 8].

Children who are born in poor family receive a little mental stimulation, and they are far more likely than their richer peers to grow up in body and mind [2, 6, 8]. In developing countries, 126 million children are living in absolute poverty. Poverty has a significant association with poor maternal education, increment of maternal stress and depression, and high levels of stressful conditions linked to violence, poor housing and lack of overall services [7, 9].

In Ethiopia, comprehensive school health and nutrition intervention are not started yet. The limited practical experience and clear understanding on how to implement as well as the lack evidence on the types of effective strategies for the country setting are among the factors that hinder implementation of school-based health and nutrition education in the country [4, 5]. Agriculture is a dominant form of livelihood in Jimma zone and only 15 % of total populations depends on non-farm jobs. However, recent evidences show that chronic food insecurity related to the escalation of food prices is pervasive among households and adolescents in the Jimma zone interfering with the school attendance and health of adolescents [6, 7]. Therefore, this study is designed to generate an evidence on the association between food insecurity and school absenteeism in the context of in Jimma Zone, South-west of Ethiopia.

## Methods

### Study setting and sampling

A school based cross-sectional study was conducted in the Jimma from October-November, 2013. Jimma Zone is one of the 20 administrative zones of Oromia Regional State with its capital Jimma town located at 350 km from Addis Ababa, Ethiopia, in the southwest direction. Jimma Zone has 18 administrative districts containing a total population of 2.5 million with the majority (94 %) living in the rural settings. The study area was stratified into urban schools and rural schools to represent a range of ecological and developmental settings. Four primary schools were selected from both urban and rural schools. Then, from each school, sections (classes) were identified and eligible study participants were randomly selected. A total of 1000 students who were permanent residents attending the school of the study area were randomly selected using their rosters as a frame. The sample was calculated using Gpower 3.0 with the following assumptions: 90.4 % an expected prevalence of poor dietary practices among food insecure school adolescents, 0.42 odds ratio of poor dietary practices among food insecure adolescents (29), margin of error of 5 % and power of 88 %. This gives a total sample size 434 and multiplied with a design effect of two. Finally, 15 % non-response was added to a final sample.

### Measurements

Structured questionnaire was used to collect data on food security, socio-demographic and economic variables. Questionnaires were prepared in English and translated to Amharic and Afan Oromo by language experts. Finally, questionnaires were retranslated back to English by a person who can speak both languages. After the recruitment of data collectors, questionnaire was pre-tested for its clarity and time required. Based on a pretest, additional adjustment was made on terminologies and the formats of the questionnaire. Data were collected by trained data collectors who were selected depending on their abilities of speaking the local language. Supervisors kept track of the field procedures and daily checked the completed questionnaires to ensure accuracy of the collected data. To avoid the possibility of measurement bias, data collectors were blinded of the study objective.

Household food insecurity was measured in the last three months using household food insecurity scales that were validated for use in developing countries [6, 7, 11] which included questions (I) the respondent worried about food (II) the household run out of food (III) reduced food variety and enforced to eat similar food (IV) reduced the amount of food intake and skipped meal (V), the respondent or another adult did not have enough to eat (VI) and felt hungry due to lack of food and stayed without food for 24 h. All “Yes” responses were coded as one and “No” responses were coded as zero. Finally, all responses were summed and dichotomized to food secure and food insecure to create an index of household food insecurity. School absenteeism was defined as any illegitimate absence from school for at least a day within the last semester, which do not include formal school closure days (national holidays or religious days).

### Data analysis

Data analyses were done using SPSS for windows version 16.0 (Chicago, Illinois) after checking for missing values and outliers. Descriptive statistics were presented using standard statistical parameters such as percentages, means and standard deviations. Multivariable logistic regression analyses were used to determine the association of school absenteeism and food insecurity with independent variables using odds ratio and 95 % of confidence intervals. Multicollinearity among independent variables was assessed using the standard errors. The standard errors for regression coefficients <2.0, as a familiar cutoff value, showed that there was no multicollinearity among independent variables. Variables that have p values ≤0.25 with dependent variables in the bivariate analyses were selected as a candidate variable for multivariable logistic regression analyses to control for associations among the independent variables.

## Results

Among eligible study participants, the majority of them (99 %) gave complete response. Bivariate analysis showed that school absenteeism was significantly associated with gender (*P* = 0.001), residence (*P* = 0.021), household head (*P* = 0.001), paternal occupation (*P* = 0.001) and age of student (*P* = 0.003) (Table 1).

**Table 1:** Socio-demographic characteristics of school adolescents and their families by school absenteeism in Jimma zone, 2013

Variables	Categories	Never absent (%)	Absent (%)	*P*-value
Household head	Father	391 (51.8)	364 (48.2)	0.001
	Mother	77 (58.8)	54 (41.2)	
	Others	22 (36.6)	35 (61.4)	
Caregivers’ marriage	Married	424 (52.2)	389 (47.8)	0.518
	Not married	81 (49.4)	83 (50.6)	
Family size	≤4	105 (47.5)	116 (52.5)	0.902
	≥5	299 (48.0)	324 (52.0)	
Student gender	Male	205 (56.6)	157 (43.4)	0.001
	Female	271 (43.2)	357 (56.8)	
Residence	Urban	244 (48.3)	261 (51.7)	0.021
	Rural	270 (55.7)	215 (44.3)	
Caregiver’s religion	Christian	169 (50.8)	164 (49.2)	0.638
	Muslim	336 (52.3)	306 (47.7)	
Mother’s age	≤29	33 (56.9)	25 (43.1)	0.160
	30–34	84 (59.6)	57 (40.4)	
	35–39	163 (50.8)	158 (49.2)	
	≥ 40	133 (48.7)	140 (51.3)	
Maternal Education	No education	163 (52.6)	147 (47.4)	0.072
	Read and write^a^	78 (60.0)	52 (40.0)	
	1^0^school (1–8)	173 (47.4)	192 (52.6)	
	2^0^school (9–12)	67 (56.8)	51 (43.2)	
	Collage & above	18 (43.9)	23 (56.1)	
Maternal job	Housewives	287 (50.9)	277 (49.1)	0.567
	Farmers	21 (46.7)	24 (53.3)	
	Others	125 (54.1)	106 (45.9)	
Paternal education	No education	58 (43.6)	75 (56.4)	0.001
	Read and write^a^	86 (67.2)	42 (32.8)	
	1^0^ school (1–8)	177 (47.6)	195 (52.4)	
	2^0^school (9–12)	99 (54.4)	83 (45.6)	
	Collage & above	63 (60.0)	42 (40.0)	
Paternal occupation	Farmers	217 (48.3)	232 (51.7)	0.035
	Government employee	93 (60.0)	62 (40.0)	
	Others	163 (53.6)	141 (46.4)	
Student age	< 14 years	384 (54.6)	319 (45.4)	0.003
	>15 years	116 (43.9)	148 (56.1)	
Student birth order	First child	136 (50.2)	135 (49.8)	0.139
	Second and above	375(52.6)	338(47.4)	

^a^who learnt adult education or attended non-formal education

**Fig. 1:**
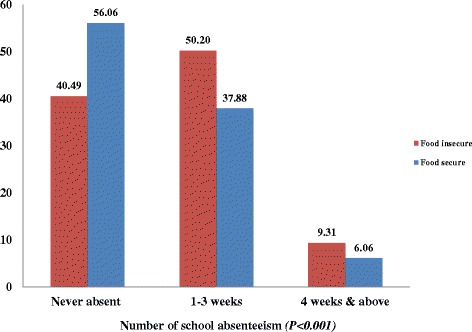
Frequency of school absenteeism by household food security status among school adolescents in Jimma zone, Ethiopia, 2013

The frequency of school absenteeism is significantly high among adolescents from food insecure households compared to those from food secure households (<0.001). A large proportion (56.06 %) of adolescents from food secure households were never absent from school compared to 40.49 % adolescents from food insecure households. Among those who abstained for more than one month and above, a relatively high proportion (9.31 %) of adolescents from food insecure households were more absent compared to adolescents from food secure households (6.1 %) (Fig. 1).

On bivariate logistic regression model household head, male gender, urban residence, age of student, household food insecurity, maternal age, seasonal food shortage and household economic status were significantly associated with adolescent school absenteeism. However, in multivariable logistic regression only household head, male gender, residence and household food insecurity were significantly associated with adolescent school absenteeism (Table 2).

**Table 2:** Factors associated with school absenteeism among school adolescents in Jimma zone, Southwest Ethiopia, 2013

Variables	Categories	School absenteeism				
		Yes	No	COR (95 % CI)	AOR (95 % CI)	*P*-value
Household head	Father	48.2	51.8	0.59 (0.34, 1.02)	0.44 (0.16, 1.16)	0.092
	Mother	41.2	58.8	0.44 (0.23, 0.83)*	0.23 (0.07, 0.72)	0.012
	Relatives	61.4	38.6	1	1	
Student sex	Male	43.4	56.6	0.58 (0.45, 0.76)**	0.64 (0.54, 0.74)	0.005
	Female	56.8	43.2	1	1	
Student residence	Urban	48.3	51.7	0.74 (0.41, 0.93)**	0.52 (0.36, 0.81)	0.003
	Rural	55.7	44.3	1	1	
Student age (in years)	< 14	45.4	54.6	0.65 (0.49, 0.87)**	0.87 (0.57, 1.36)	0.540
	>15	56.1	43.9	1	1	
Paternal occupation	Farmer	51.7	48.3	1.24 (0.92, 1.66)	1.02 (0.48, 2.16)	0.944
	Government worker	40.0	60.0	0.77 (0.52, 1.14)	0.64 (0.35, 1.15)	0.143
	Others^a^	46.4	53.6	1	1	
Maternal age (in years)	≤29	43.1	56.9	0.72 (0.41, 1.27)	0.80 (0.38, 1.68)	0.586
	30–34	40.4	59.6	0.65 (0.43, 0.97)*	0.69 (0.40, 1.18)	0.177
	35–39	49.2	50.8	0.92 (0.67, 1.27)	0.82 (0.52, 1.27)	0.398
	≥40	51.3	48.7	1	1	
Food secure status	Food insecure	59.5	40.5	1.88 (1.39, 2.52)**	2.81 (1.70, 4.76)	0.001
	Food secure	43.9	56.1	1	1	
Seasonal food shortage	No	37.4	62.6	0.62 (0.37, 0.96)*	0.30 (0.14, 2.05)	0.093
	Yes	49.1	50.9	1	1	
Hunger at school	No	47.1	52.9	0.74 (0.46, 1.18)	0.82 (0.33, 2.04)	0.661
	Yes	54.7	45.3	1	1	
Student illness	No	47.1	52.9	0.85 (0.65, 1.12)	1.36 (0.90, 2.06)	0.150
	Yes	51.0	49.0	1	1	
Household size	<5	47.3	52.7	0.97 (0.33, 0.99)*	1.16 (0.54, 2.60)	0.701
	≥5	48.0	52.0	1	1	
Household Economic Status	Low	54.1	45.9	1.47 (1.01, 2.17)*	1.14 (0.59, 2.16)	0.701
	Medium	44.6	55.4	1.01 (0.71, 1.41)	0.81 (0.48, 1.37)	0.426
	High	44.5	55.5	1	1	

*COR* Crude Odd ratio, *AOR* Adjusted Odd Ratio

*Significant at <0.05, **significant at <0.001, ^a^Daily laborer, merchant, non-government worker

Findings of multivariable logistic regression analyses showed that adolescents from food insecure households were 81 % more absent from school [AOR = 2.81 (1.70, 4.76)] compared to their peers from food secure households.

This study showed that adolescents from female-headed families were 77 % less likely absent from school [AOR = 0.23 (0.07, 0.72)] compared to their peers from families headed by their relatives. Adolescents from urban areas were 48 % less likely absent from school [AOR = 0.52 (0.36, 0.81)] compared to rural adolescents. Similarly, male adolescents were 34 % less likely absent [AOR = 0.64 (0.54, 0.74)] from school compared to female adolescents.

Household food insecurity was significantly associated with maternal educational status, household income, having livestock and the economic status of their family (Table 3). Maternal education status was significantly associated with household food insecurity in which women who attended only primary school were 20 % more likely food insecure household [AOR = 4.20 (1.22, 17.33)] compared to women who attended college and above.

**Table 3:** Factors associated with household food insecurity among school adolescents in Jimma zone, Southwest Ethiopia, 2013

	Food secure household					
Variables	Category	No	Yes	COR (95 % CI)	AOR (95 % CI)	*P*-value
Household head	Father	31.2	68.8	1.24 (0.66, 2.28)	1.80 (0.56, 5.81)	0.326
	Mother	23.4	76.6	0.84 (0.42, 1.69)	2.29 (0.55, 9.65)	0.257
	Relatives	26.8	73.2	1	1	
Maternal education	No education	30.9	69.1	0.86 (0.41, 0.79)*	3.05 (0.78, 11.95)	0.109
	Can read and write	33.3	66.7	0.97 (0.44, 2.15)	2.26 (0.57, 8.93)	0.064
	Primary school(1–8)	40.1	59.9	1.29 (0.62, 2.69)	4.20 (1.22, 17.33)	0.024
	Secondary school (9–12)	39.4	60.6	1.66 (0.72, 3.85)	5.36 (1.25, 22.10)	0.244
	Collage and above	34.1	65.9	1	1	
Maternal occupation	Housewives	28.6	71.4	1.18 (0.83, 1.68)	0.78 (0.39, 1.55)	0.476
	Farmers	24.3	75.7	0.61 (0.31, 1.19)	0.41 (0.14, 1.23)	0.112
	Others^a^	25.3	74.7	1	1	
Type of house	Self-build house	32.4	67.6	1.81 (1.13, 2.90)	1.80 (0.70, 4.62)	0.224
	Government house	25.9	74.1	1.31 (0.74, 2.31)	0.94 (0.32, 2.77)	0.911
	Rented house	21	79	1	1	
Caregivers’ residence	Urban	24.3	75.7	0.95 (0.71, 1.27)	1.34 (0.42, 4.26)	0.620
	Rural	25.2	74.8	1	1	
Caregivers’ Sex	Male	22.3	77.7	0.94 (0.69, 1.26)	0.89 (0.54, 1.47)	0.653
	Female	23.3	76.7	1	1	
Fathers’ job	Farmers	29	71	1.07 (0.73, 1.57)	0.83 (0.28, 2.44)	0.730
	Government employee	30.8	69.2	1.16 (0.71, 1.89)	0.90 (0.36, 2.28)	0.831
	Private Organization	42.6	57.4	1.95 (1.08, 3.55)	1.25 (0.47, 3.30)	0.655
	Others	27.7	72.3	1	1	
Fathers education	No education	22.2	77.8	0.53 (0.29, 0.95)*	0.86 (0.25, 2.94)	0.814
	Can read and write	31	69	0.83 (0.45, 1.53)	1.11 (0.30, 4.09)	0.879
	Primary school(1–8)	33.4	66.6	0.93 (0.55, 1.56)	0.78 (0.27, 2.27)	0.653
	Secondary school (9–12)	38.3	61.7	1.15 (0.63, 2.07)	1.16 (0.40, 3.33)	0.785
	Collage and above	35.1	64.9	1	1	
Caregivers’ illness	Yes	31.2	68.8	1.98 (1.45, 2.70)**	1.41 (0.83, 2.40)	0.202
	No	18.7	81.3	1	1	
Family economic situation	Self-sufficient	2.8	97.2	0.06 (0.03, 0.10)**	0.17 (0.06, 0.51)	0.001
	Not self-sufficient	31.3	68.7	1		
Family size	≤4	21.6	78.4	0.78 (0.55, 1.10)	0.93 (0.51, 1.69)	0.814
	≥5	26	74	1	1	
Livestock ownership	Have livestock	2.5	97.5	0.02 (0.01, 0.04)**	0.09 (0.01, 0.15)	0.001
	Have no livestock	51.6	48.4	1		
Household economic status	Low	19.3	80.7	0.58 (0.37, 0.93)*	1.39 (1.18, 2.83)	0.014
	Medium	24.6	75.4	0.79 (0.53, 1.17)	0.68 (0.36, 1.30)	0.240
	High	29.3	70.7	1	1	
Marital Status	Married	25.5	74.5	0.92 (0.66, 1.28)	0.64 (0.36, 1.13)	0.125
	Not Married	27	73	1	1	

*COR* Crude Odd ratio, *AOR* Adjusted Odd Ratio

*Significant at <0.05, **significant at <0.001,

^a^Daily laborer, government and non-government employee

Source of household income was significantly associated with household food insecurity were households who did not depends on others to generate their own income were 17 % less likely food insecure [AOR = 0.17 (0.06, 0.51)] compared to households who depends on others. Adolescents whose their families have livestock were 9 % less likely food insecure [AOR = 0.09 (0.02, 0.15)] compared to adolescents whose their family had no livestock.

The household economic status was also significantly associated with household food security status in which poor households were 39 % more food insecure [AOR = 1. 39 (1.18, 2.83)] than households from high economic status.

## Discussion

Findings of this study showed that school absenteeism among school adolescents were significantly high among food insecure households. There are several pathways for this link between food insecurity and poor school attendance. Food insecure adolescents may not be able to go school due to illness, lack of access to health service and food provision at breakfast. Evidences from different studies showed that health and nutrition problems are major barriers to educational access and academic performance in low-income countries [4, 12]. Both food insecurity and lack of education are the most common deprivations that pose a serious challenge to the sustainable development goals of Education for all in developing countries [4, 5, 12, 13]. Studies from countries like Canada, US and Venezuela showed that food insecurity strongly affects school activity, academic performance and cognitive development which are also common predictors of school absenteeism [14, 15].

School absenteeism was significantly associated with gender differences in which males were less absent from school compared to females. This might be due to cultural and socio-economic constraints which enforced females to take house work responsibility and child care. Studies from different socio-cultural backgrounds also showed limited access to education and decision-making, burden of unpaid work and gender-based violence are common contributors to females’ school absenteeism. FAO reported that a large number of the world’s poor people are a rural resident where agriculture is the main source of income. However, rural women have limited access to land and gender inequalities in control of livelihood assets limit women’s food production [4, 16, 17].

This study showed that rural students were less school attendant compared to their peers from urban. This might be due to the socio-cultural barriers like, harmful traditional practices and lack of awareness about the ultimate benefit of education, wrong perception of about disabled children and females. School availability, facility distribution and distance can also be barriers to school attendance in the rural areas. Studies also showed that a large proportion of rural households in developing countries like Ethiopia rely primarily on their own food production [12, 17]. Poor infrastructure and transportation, inability to import food and low foreign exchange severely limit people’s ability to the school [12, 16, 17].

Adolescents from female-headed households were less likely absent from school compared to adolescents from households headed by relatives. This difference might be due to mothers’ care for their children compared to the children who are not directly under the supervision of their mothers. Studies also showed that due to economic constraints and other social factors the students will take additional responsibilities at home on the top of their school activities like keeping cattle, fetching water and wood-fire collection. These are common problems in developing countries like Ethiopia and it is more severe, especially if young adolescents are not directly under supervision of their families. These burdens have also a significant impact on school readiness, engagement, attendance and academic performance [18, 19].

Our results imply that having a source of income had a significant input to household food security status which has consistency with the study done in Kenya [15]. Additionally, the finding of this study indicated that households who had livestock were less food insecure compared to those who had no livestock. The study from Pakistan and Haiti similarly showed that the incidence of poverty is less among those who had livestock, which has financial value for farmers to purchase food for their families [14, 20].

Maternal education was also strongly associated with household food security status. Students whose mothers attended only primary school were more food insecure household than whose mothers attended college and above. This might be due to the fact that working mothers contribute to total household income and educated mothers are more likely to be aware of nutrition, hygiene and health care. Studies from Nigeria and other African countries also showed maternal education have strong relationship with food security status [11, 21]. Educational status of women clearly has an essential element to reduce rural food insecurity. Findings from Mozambique showed that education for mothers is a key to tackle children food insecurity within rural households. The relationship of household income and food security has a consecutive association between household expenditure for food and dietary intake that leads to household food security [22–25].

Household food insecurity was significantly associated with the household economic status where poor households were more food insecure than wealthy households. Different studies also showed that there is a significant difference of monthly household income and household expenditure for food between food-insecure and food-secure households. In addition, food consumption results from a multi-factorial behaviour influenced by the affordability and availability of the food, prevalent culture, religion and food eating habits [11, 13].

The results of this study have a significant contribution for researchers and national nutrition programs in the improvement of school based health and nutrition education to achieve the sustainable development goals of inclusive and equitable quality education and lifelong learning opportunities for all. However, the cross-sectional nature of this data limits to draw any causal conclusions. There might be also recall bias as the caregivers or adolescents could forget their past dietary intake, but intensive training was given for data collectors on how to probe caregivers and adolescents to remember their dietary practices.

## Conclusions

Our study showed that school absenteeism is positively associated with household food insecurity, female gender and rural residence. Household food insecurity was significantly associated with household economic status, having livestock and maternal education. Therefore, there is a need of multisectoral efforts among different sectors to improve household earning capacity and socio-economic status to tackle household food insecurity which has a strong linkage with that of adolescent school absenteeism.

## Abbreviations

AOR: Adjusted odds ratio
CI: Confidence intervals
COR: Crude odds ratio

## References

[1] Food and Agriculture Organization of the United Nations. The State of Food Insecurity in the World: The multiple dimensions of food security. Rome; 2013. http://www.fao.org/docrep/018/i3434e/i3434e.pdf. Accessed Oct 2014.

[2] Levinger B. Nutrition, health and learning: Current issues and trends. School Nutrition and Health Network Monograph Series, #1. Massachusetts, USA; 1992. http://pdf.usaid.gov/pdf_docs/PNABQ180.pdf. Accessed June 2014.

[3] Meredith H, Cuba S, Weiss I, Donofrio G, Cook J. Too Hungry to Learn: Food Insecurity and School Readiness. Boston; 2013. http://www.childrenshealthwatch.org/publication/too-hungry-to-learn/. Accessed Nov 2015.

[4] Belachew T, Hadkley C, Lindstrom D, Gebrenmariam A, Lachat C, Kolsteren P (2011). Food insecurity, school absenteeism and educational attainment of adolescents in Jimma Zone Southwest Ethiopia: a longitudinal study. Nutr J.

[5] Winicki J, Jemison K (2003). Food insecurity and hunger in the kindergarten classroom: Its effects on learning and growth. Contemp Econ Policy.

[6] Belachew T, Hadkley C, Lindstrom D, Gebrenmariam A, Lachat C, Kolsteren P (2012). Predictors of chronic Food Insecurity among adolescents in southwest Ethiopia. BMC Public Health.

[7] Hadley C, Stevenson EG, Tadesse Y, Belachew T (2012). Rapidly rising food prices and the experience of food insecurity in urban Ethiopia: Impacts on health and well-being. Soc Sci Med.

[8] Eming M (2014). Addressing and mitigating vulnerability across the life cycle: the case for investing in early childhood.

[9] Muro P, Burchi F. Education for Rural People and Food Security: A Cross Country Analysis. Rome; 2007. http://www.fao.org/docrep/010/a1434e/a1434e00.htm. Accessed June 2015.

[10] Matheson D, Varady J, Varady A, Killen J (2002). Household food security and nutritional status of Hispanic children in the fifth grade. Am J Clin Nutr.

[11] United Nations Economic and Social Council. Status of Food Security in Africa. Addis Ababa; 2012. http://www.uneca.org/sites/default/files/uploaded-documents/CFSSD/CFSSD8/3._cfssd-8-0032-orestatus_of_food_security_in_africa_2012.pdf

[12] Moyi P (2013). An examination of primary school attendance and completion among secondary school Age adolescents in post-conflict Sierra Leone. Res Comp Int Educ.

[13] World Health Organization. The Status of Poverty and Food Security in Egypt: Analysis and Policy Recommendations. Cairo; 2013. http://documents.wfp.org/stellent/groups/public/documents/ena/wfp257467.pdf. Accessed Oct 2014.

[14] Roustit C, Hamelin A, Grillo F, Martin J, Chauvin P (2010). Food Insecurity: Could school food supplementation help break cycles of intergenerational transmission of social inequalities?. Pediatrics.

[15] Diana F, Edward A, Sonya J (2005). Food insecurity affects school Children’s academic performance, weight gain, and social skills. J Nutr.

[16] Mehra R, Rojas M (2008). Women, food security and agriculture in a global marketplace.

[17] Food and Agriculture Organization. Gender dimensions of agricultural and rural employment: Differentiated pathways out of poverty Status, trends and gaps. Rome; 2010. http://www.fao.org/docrep/013/i1638e/i1638e.pdf

[18] Huebler F. Child labour and school attendance: Evidence from MICS and DHS surveys. New York; 2008. http://www.unicef.org/protection/Child_labour_school_FHuebler_2008. Accessed July 2014.

[19] Bernal J, Frongillo E, Herrera H, Rivera J (2014). Food insecurity in children but Not in their mothers is associated with altered activities, school absenteeism, and stunting. J Nutr.

[20] Ali A, Khan M (2013). Livestock ownership in ensuring rural household food security in Pakistan. J Anim Plant Sci.

[21] Ajao K, Ojofeitimi E, Adebayo A, Fatusi A, Afolabi O (2010). Influence of family size, household food security status, and child care practices on the nutritional status of under-five children in Ile-Ife, Nigeria. Afr J Reprod Health.

[22] Burchi F, Muro P (2005). Natural Resources Management and Environment Department (NR) Education for Rural People Initiative. Reducing Children’s Food Insecurity through Primary Education for Rural Mothers: The case of Mozambique.

[23] Shariff Z, Khor G (2008). Household food insecurity and coping strategies in a poor rural community in Malaysia. Nutr Res Pract.

[24] Frongillo EA, De Onis M, Hanson KM (1997). Socioeconomic and Demographic Factors Are Associated with Worldwide Patterns of Stunting and Wasting of Children. J Nutr.

[25] Naser I, Jalil R, Muda W, Nik W, Shariff Z, Abdullah M (2014). Association between household food insecurity and nutritional outcomes among children in North-eastern of Peninsular Malaysia. Nutr Res Pract.

